# Does Having a Usual Primary Care Provider Reduce Polypharmacy Behaviors of Patients With Chronic Disease? A Retrospective Study in Hubei Province, China

**DOI:** 10.3389/fphar.2021.802097

**Published:** 2022-01-21

**Authors:** Jia Wang, Zhanchun Feng, Zhongxin Dong, Wanping Li, Chaoyi Chen, Zhichun Gu, Anhua Wei, Da Feng

**Affiliations:** ^1^ School of Pharmacy, Tongji Medical College of Huazhong University of Science and Technology, Wuhan, China; ^2^ School of Medicine and Health Management, Tongji Medical College of Huazhong University of Science and Technology, Wuhan, China; ^3^ Renji Hospital, School of Medicine, Shanghai Jiao Tong University, Shanghai, China; ^4^ Department of Pharmacy, Tongji Hospital, Tongji Medical College, Huazhong University of Science and Technology, Wuhan, China

**Keywords:** polypharmacy, chronic disease, usual primary care providers, propensity score weight, service utilization

## Abstract

**Background:** Within China's hierarchical medical system, many patients seek medical care in different hospitals independently without integrated management. As a result, multi-hospital visiting is associated with fragmented service utilization and increased incidence of polypharmacy behaviors, especially for patients with chronic disease. It has been confirmed that factors from the perspective of patients may cause polypharmacy behaviors in Chinese community patients; whether having a usual primary care provider for chronic disease patients could reduce the polypharmacy behaviors and the effect size remains unanswered, and that is what our study aimed to answer.

**Methods:** Our study adopted a cluster sampling method to select 1,196 patients with hypertension or diabetes and measured some information about them. The propensity score weighting method was adopted to eliminate the influence of confounding bias, and then a multivariate logistic regression model was conducted to test the relationship between having a usual primary care provider and polypharmacy behaviors.

**Results:** Patients without usual primary care providers were significantly correlated with polypharmacy behaviors (OR = 2.40, 95%CI: 1.74–3.32, *p* < 0.001), and the corresponding marginal effect is 0.09 (95%CI: 0.06–0.12). Patients who suffer from two kinds of diseases (OR = 3.05, 95%CI: 1.87–5.10, *p* < 0.001), with more than three kinds of diseases (OR = 21.03, 95%CI: 12.83–35.65, *p* < 0.001), with disease history of 20 years and above (OR = 1.66, 95%CI: 1.14–2.42, *p* = 0.008), who communicate frequently with doctors (OR = 3.14, 95%CI: 1.62–6.19, *p* < 0.001), alcoholic patients (OR = 2.14, 95%CI: 1.08–4.19, *p* = 0.027), who used to have meat-based food (OR = 1.42, 95%CI: 1.00–2.00, *p* = 0.049), and have vegetarian-based diet (OR = 1.42, 95%CI: 1.00–2.00, *p* = 0.049) are more likely to have polypharmacy behaviors, while patients aged between 65 and 75 years (OR = 0.50, 95%CI: 0.33–0.77, *p* = 0.020), used to be brain workers (OR = 0.67, 95%CI: 0.45–0.99, *p* = 0.048), with disease history between 10 and 20 years (OR = 0.56, 95%CI: 0.37–0.83, *p* = 0.005), have had adverse drug reactions (OR = 0.64, 95%CI: 0.45–0.93, *p* = 0.019), and participated in medical insurance for urban and rural residents (OR = 0.35, 95%CI: 0.21–0.58, *p* < 0.001) were less likely to have polypharmacy behaviors.

**Conclusion:** The results suggest that having a usual primary care provider may reduce the incidence of having polypharmacy behaviors; we can take intervention measures to promote establishing a long-term relationship between patients and primary care providers.

**Systematic Review Registration:** [website], identifier [registration number]

## Introduction

Chronic non-communicable diseases (NCDs) are a kind of disease with insidious onset, long incubation period, long and slow course, uncured, lack of convinced evidence of biological etiology, and no clear indications for treatment. As the population ages, NCDs have become one of the greatest threats to public health. According to the World Health Statistics Report 2021 released by the WHO, 7 of the top 10 causes of death in 2019 were chronic diseases. In 2000, 60.8% of patients died from NCDs, and the proportion rose to 73.6% in 2019. Demographic changes such as the aging of the population promote the development of multimorbidity of chronic conditions (Xu et al., 2017). Multimorbidity always leads to polypharmacy behaviors among patients with chronic diseases. Polypharmacy behaviors have been considered to be an increasingly serious public health problem worldwide, especially among the elderly (Payne and Avery, 2011; Wastesson et al., 2018). The vast majority of studies defined taking five or more drugs at the same time as polypharmacy behaviors (Kaufman et al., 2002; Fialová, 2005; Payne and Avery, 2011; Mortazavi et al., 2016; Masnoon et al., 2017; Wastesson et al., 2018). A study in United States showed that 29.0% of elderly people used at least five prescription drugs. Another survey on community residents showed that 37.1% of men aged 75 to 85 years and 36.0% of women took five or more prescription drugs at the same time (Hoel et al., 2021). In the 2016 UK Health Survey, 56.0% of people aged ≥85 years took five or more drugs (UK.GOV, 2021), while about 9.0% of people aged 45–54 years took the same. A review report of polypharmacy behaviors found that the incidence of polypharmacy behaviors in various countries ranged from 38.1% to 91.2% (Jokanovic et al., 2015). Polypharmacy behaviors are becoming increasingly popular.

It has been reported that patients who take five medications have at least one serious medication problem (Strand et al., 1990; Hanlon et al., 2006). Polypharmacy behaviors are associated with an increase in adverse drug reactions, lower compliance, inappropriate drug therapies, and higher medical expenditure (Field et al., 2001; Goulding, 2004; Lau et al., 2005; Payne and Avery, 2011; Wastesson et al., 2018). Some other studies have also found that polypharmacy behaviors are associated with rehospitalization, falls, death, and poor health outcomes (Bushardt et al., 2008; Cooney and Pascuzzi, 2009; Dhalwani et al., 2017; Leelakanok et al., 2017). It also increases the economic burden of the health system (Fulton and Riley Allen, 2005).

The occurrence of polypharmacy behaviors is related to many factors. From the patients' perspective, demographic factors such as age, education, health behaviors (smoking, drinking, exercise, and daily eating habits) have significantly related to polypharmacy behaviors. In addition, disease-related factors have also been confirmed to cause polypharmacy behaviors in patients with chronic diseases. Many studies have found that the type of disease, the number of diseases, and the severity of the disease will affect the occurrence of polypharmacy behaviors in patients with chronic diseases (Bronskill et al., 2012; Liu et al., 2021). Medical cause has also been confirmed to be related to patients' polypharmacy behaviors. Inpatients had an average of two more drugs when compared with outpatients (Betteridge et al., 2012; Viktil et al., 2012). The disorder of multi-institution medical treatment and the lack of comprehensive management of patients' multi-institution medical treatment leads to problems such as repeated prescription, excessive prescription, misuse of prescription, and prescribing cascade, thus leading to polypharmacy behaviors (O'Connor et al., 2012). Moreover, from the care provider perspective, patients were consulted in different medical institutions and departments, resulting in doctor–patient communication not being smooth, fragmentation of medication regimen, and increased risk of polypharmacy behaviors. Some patients prefer to seek healthcare in different hospitals, which could not provide integrated medication recommendations and always resulted in repeat or unnecessary medication. However, few studies measured the factors that influenced polypharmacy behaviors in terms of service utilization and usual primary health care in China; whether having a usual primary care provider for chronic disease patients could reduce the polypharmacy behaviors and the effect size remains unanswered. On the whole, there is a research gap in exploring the relationship between usual care provider and polypharmacy behaviors. Our study aimed to explore the relationship between the usual primary health care and polypharmacy behaviors of patients with chronic diseases and quantify the impact effect.

## Methods

### Study Settings and Sampling

The data were collected by a cluster sampling method from April to June 2021, in which we first selected four cities (Zhijiang, Qianjiang, Yichang, and Wuhan) from Hubei province, China. Wuhan and Yichang were taken as urban sample areas, while Zhijiang and Qianjiang were taken as rural sample areas. Three streets or towns were randomly selected from each city (a total of 12), and every street or town needed to collect 100 patients diagnosed with hypertension or diabetes, which can be managed by primary health care providers. Exclusion criteria included those who had not been taking medicine for more than 3 months, those who were younger than 18 years, those who cannot express themselves clearly, those who were unwilling to cooperate with this investigation, those who were severely ill and cannot complete the questionnaire, and those diagnosed with acute complications. In total, 1,205 participants joined the survey; 9 of them were excluded due to lack of information.

### Variables and Measurement

The primary predictor of interest in this study was whether a patient reported having a usual primary care provider. In this study, the usual primary care providers were determined by asking the patients “Which of the following institutions have you frequently visited in the past 3 months?” Respondents who frequently visited primary health care institutions were considered to have primary health care providers, and the others were considered to have no primary health care providers.

The dependent variable was whether patients had polypharmacy behaviors; they were considered to have polypharmacy behaviors if they took five or more drugs per day, and other patients were regarded as non-polypharmacy.

Confounding variables included demographic characteristics, clinical conditions, medical treatment behavior data, health information data of patients with chronic diseases, and medication knowledge of patients.

Demographic data included the patient's domicile, gender, age, education level, annual income, job type, and residence status. Job type is classified into brain workers and manual workers. Residence status is divided into live alone and not live alone.

Clinical conditions included the number of chronic diseases, disease history, adverse drug reactions (ADRs), and severity of disease. Disease history is calculated based on the question “when were you diagnosed with chronic disease?”

Patients' medical treatment behaviors data included whether in hospital in the past year, type of medical insurance, the frequency of communication with doctors, etc. The type of medical insurance was divided into employee health insurance and urban and rural resident medical insurance. The frequency of communication with doctors is classified as none, rarely, occasionally, often, and always.

Health information data included smoking status, drink status, physical activity, and whether people with chronic diseases had their blood pressure or blood sugar checked regularly.

Patient knowledge was evaluated through administration of a questionnaire adapted from the study by McPherson et al. (Mcpherson et al., 2008; Okuyan et al., 2013); the median was taken as the critical value to divide the total score of medication knowledge into high and low score groups of medication knowledge.

### Statistical Analysis

A descriptive analysis of sample characteristics was performed on SPSS 24.0, and the other statistical analyses were performed on R Commander Version 4.04, which is a graphical user interface for R software. Multivariate logistic regression analysis was conducted to model the association between having a usual primary care provider and participants' polypharmacy behaviors. Propensity-score weighting approach was applied to adjust for the observed difference in characteristics between those having a usual primary care provider and those not having and therefore could better tease out the net influence of having a usual primary care provider on patients' polypharmacy behaviors. Odds ratios (ORs), marginal effects, and their 95% confidence intervals were reported to indicate the relationship between the two variables. Statistical significance was defined as a two-tailed *p*-value <0.05 in all analyses.

### Ethics Statement

The study was conducted in accordance with the Declaration of Helsinki. Written informed consent was obtained from all patients. This study was approved by the Medical Ethics Committee of Tongji Medical College of Huazhong University of Science and Technology, and the approval number is 2020 (S223).

## Results

### Interviewee Information

As shown in Table 1, the characteristics of a total of 1,205 patients with chronic diseases who participated were collected in this survey, of which 1,196 completed questionnaires. About 71.0% (849/1,196) were 65 years old and above; the youngest patient was 26 years old, the oldest patient was 92 years old, and the average age was 68.55 years old. About 58.0% (693/1,196) were female. Approximately 48.3% (578/1,196) were living in the urban area. About 62.6% (749/1,196) were manual workers; 10.4% lived alone. In general, there were two kinds of chronic diseases per capita and three kinds of medication per capita taken. Two hundred fifty-two patients (21.1%) had polypharmacy behaviors. Adverse reactions occurred in 242 patients (20.2%), and 399 (33.7%) patients frequently visited primary medical institutions.

**TABLE 1:** Characteristics of the study population.

	Non-polypharmacy N (%)	Polypharmacy N (%)	Χ^2^	*p*
A usual primary care provider				
Yes	352 (37.3)	47 (18.7)	31.077	<0.001
No	592 (62.7)	205 (81.3)		
Gender				
Male	397 (42.1)	106 (42.1)	0.000	0.998
Female	547 (57.9)	146 (57.9)		
Age (years)				
18–65	280 (29.7)	67 (26.6)	5.249	0.072
66–75	508 (53.8)	124 (49.2)		
>75	156 (16.5)	61 (24.2)		
Domicile				
Urban area	436 (46.2)	142 (56.3)	8.226	0.004
Rural area	508 (53.8)	110 (43.7)		
Education				
Primary school	437 (46.3)	106 (41.9)	5.723	0.126
Middle school	285 (30.2)	76 (30.0)		
High school	174 (18.5)	49 (19.4)		
University	47 (5.0)	22 (8.7)		
Job				
Manual worker	611 (64.7)	138 (54.8)	8.435	0.004
Brain worker	333 (35.3)	114 (45.2)		
Residence status				
Live alone	93 (9.9)	31 (12.3)	1.285	0.257
Not live alone	851 (90.1)	221 (87.7)		
Annual income per year				
0–9,999	276 (29.2)	67 (26.6)	0.683	0.711
10,000–50,000	310 (32.8)	86 (34.1)		
>50,000	358 (37.9)	99 (39.3)		
Number of diseases				
1	445 (47.1)	24 (9.5)	134.176	0.000
2	494 (52.3)	216 (85.7)		
≥3	5 (0.5)	12 (4.8)		
Disease history/year				
0–10	497 (52.6)	104 (41.3)	14.249	0.001
11–20	254 (26.9)	71 (28.2)		
>20	193 (20.4)	77 (30.6)		
Adverse disease reaction				
No	783 (82.9)	171 (67.9)	28.054	<0.001
Yes	161 (17.1)	81 (32.1)		
Severity of disease				
Mild	433 (45.9)	48 (19.0)	97.158	0.000
Moderate	386 (40.9)	111 (44.0)		
Severe	125 (13.2)	93 (36.9)		
Medical insurance				
Employee health insurance	367 (38.9)	120 (47.6)	6.297	0.012
Resident health insurance	577 (61.1)	132 (52.4)		
Hospitalization				
Yes	291 (30.8)	141 (56.0)	54.422	<0.001
No	653 (69.2)	111 (44.0)		
Communication frequency				
No	127 (13.5)	22 (8.7)	22.421	<0.001
Rare	291 (30.8)	64 (25.4)		
Occasionally	180 (19.1)	50 (19.8)		
Often	276 (29.2)	75 (29.8)		
Always	70 (7.4)	41 (16.3)		
Drink				
Never	733 (77.6)	214 (84.9)	7.945	0.047
Occasionally	115 (12.2)	16 (6.3)		
Often	29 (3.1)	7 (2.8)		
Always	67 (7.1)	15 (6.0)		
Smoking				
Never	685 (72.6)	195 (77.4)	7.459	0.024
Have quit smoking	105 (11.1)	33 (13.1)		
Smoking	154 (16.3)	24 (9.5)		
Diet				
Balance	522 (55.3)	115 (45.6)	7.925	0.019
Mainly meat	65 (6.9)	18 (7.1)		
Mainly vegetarian	357 (37.8)	119 (47.2)		
Exercise				
Never	109 (11.5)	43 (17.1)	8.402	0.038
Occasionally	166 (17.6)	52 (20.6)		
Often	193 (20.4)	50 (19.8)		
Always	476 (50.4)	107 (42.5)		
Blood pressure measurement				
Irregular	178 (18.9)	33 (13.1)	4.543	0.033
Regular	766 (81.1)	219 (86.9)		
Knowledge of medication				
Low	482 (51.1)	125 (49.6)	0.169	0.681
High	462 (48.9)	127 (50.4)		

### Propensity Score Weighted Results

Standardized mean difference is an indicator used to evaluate the balance between the experimental group and the control group before and after weighting, using a threshold of 0.10 to indicate imbalance. As shown in Table 2, after propensity score weighting, covariates with large deviations in the original data are balanced, and the overall distribution of the data is more balanced.

**TABLE 2:** The standardized mean difference of each covariable before and after weighting.

Variable	Standardized mean difference	
	Before weight	After weight
Gender	−0.09	0.09
Age	0.27	0.00
Domicile	−1.07	0.06
Education	0.53	0.00
Job	0.58	0.04
Residence status	0.00	0.11
Annual income	0.63	0.06
Number of diseases	0.29	0.00
Disease history	0.29	0.05
Adverse disease reaction	−0.16	−0.09
Severity of disease	0.49	−0.11
Medical insurance	−0.90	−0.07
Hospitalization	−0.18	0.11
Communication frequency	−0.3	−0.05
Drink	−0.08	−0.05
Smoking	−0.03	−0.07
Diet	−0.26	−0.06
Exercise	−0.04	−0.05
Regular blood pressure measurement	−0.10	−0.07
Knowledge of medication	0.18	−0.03

### Regression Analysis Results

Tables 3, 4 present the results from multivariate logistic regressions and propensity scores weighting regressions. In this model, we calculated both ORs and marginal effects of each variable. The results of the weighted regression are different from the results of the multivariate logistic regression. The multivariate logistic regression underestimates the impact of having a usual primary care provider on the patient's multiple medication behaviors due to crowd confounding factors. Result from the propensity scores weighted regression showed that having no usual primary care providers was significantly associated with the odds of polypharmacy behaviors (OR = 2.40, 95%CI: 1.74–3.32, *p* < 0.001), and the corresponding marginal effect was 0.09 (95% CI: 0.06–0.12), indicating that patients having a usual primary care provider are less likely to have polypharmacy behaviors.

**TABLE 3:** ultivariate logistic regression before and after propensity score weighting.

Variable	Before weighting		After weighting	
	OR (95%CI)	*p*	OR (95%CI)	*p*
A usual primary care provider				
Yes				
No	1.5 (0.98–2.31)	0.065	2.40 (1.74–3.32)	<0.001
Gender				
Male				
Female	0.76 (0.48–1.21)	0.248	1.32 (0.87–2.03)	0.192
Age (years)				
18–65				
66–75	0.65 (0.42–1.01)	0.053	0.50 (0.33–0.77)	0.002
>75	0.97 (0.56–1.68)	0.914	1.03 (0.62–1.69)	0.921
Domicile				
Urban area				
Rural area	0.47 (0.25–0.89)	0.02	1.21 (0.68–2.17)	0.523
Education				
Primary school				
Middle school	1.24 (0.79–1.97)	0.350	1.13 (0.72–1.76)	0.593
High school	1.41 (0.78–2.55)	0.256	1.45 (0.84–2.50)	0.184
University	2.48 (1.09–5.6)	0.030	1.70 (0.81–3.57)	0.161
Job				
Manual worker				
Brain worker	1 (0.66–1.53)	0.992	0.67 (0.45–0.99)	0.048
Residence status				
Live alone				
Not live alone	1.06 (0.61–1.86)	0.840	1.41 (0.85–2.37)	0.191
Annual income				
0–9,999				
10,000–50,000	1.14 (0.7–1.85)	0.602	1.06 (0.67–1.69)	0.809
>50,000	1 (0.57–1.77)	0.991	1.28 (0.77–2.15)	0.345
Number of diseases				
1				
2	3.35 (2.02–5.71)	<0.001	3.05 (1.87–5.10)	<0.001
≥3	14.01 (8.34–24.39)	<0.001	21.03 (12.83–35.65)	<0.001
Disease history/year				
0–10				
11–20	0.85 (0.56–1.28)	0.435	0.56 (0.37–0.83)	0.005
>20	1.12 (0.73–1.72)	0.603	1.66 (1.14–2.42)	0.008
Adverse disease reaction				
No				
Yes	0.81 (0.54–1.21)	0.294	0.64 (0.45–0.93)	0.019
Severity of disease				
Mild				
Moderate	1.47 (0.96–2.25)	0.079	1.36 (0.90–2.09)	0.150
Severe	2.3 (1.38–3.82)	0.001	1.63 (1.00–2.67)	0.049
Medical insurance				
Employee health insurance				
Resident health insurance	1.01 (0.55–1.82)	0.975	0.35 (0.21–0.58)	<0.001
Hospitalization				
No				
Yes	0.54 (0.38–0.77)	<0.001	0.83 (0.60–1.15)	0.260
Communication frequency				
No				
Rare	1.39 (0.76–2.64)	0.296	1.06 (0.62–1.85)	0.838
Occasionally	1.41 (0.73–2.77)	0.313	1.14 (0.62–2.10)	0.673
Often	1.44 (0.77–2.8)	0.265	1.19 ((0.68–2.14)	0.561
Always	3.17 (1.51–6.82)	0.003	3.14 (1.62–6.19)	<0.001
Drink				
Never				
Occasionally	0.53 (0.26–1.02)	0.066	0.69 (0.36–1.28)	0.254
Often	1.26 (0.42–3.45)	0.663	1.12 (0.38–3.00)	0.828
Always	1.42 (0.66–2.94)	0.354	2.14 (1.08–4.19)	0.027
Smoking				
Never				
Have quit smoking	0.83 (0.45–1.51)	0.555	1.36 (0.81–2.28)	0.245
Smoking	0.58 (0.3–1.06)	0.084	0.60 (0.32–1.10)	0.105
Diet				
Balance				
Mainly meat	1.78 (0.88–3.48)	0.098	2.01 (1.05–3.73)	0.030
Mainly vegetarian	1.67 (1.16–2.41)	0.006	1.42 (1.00–2.00)	0.049
Exercise				
Never				
Occasionally	1.29 (0.72–2.32)	0.387	1.20 (0.70–2.05)	0.504
Often	1.01 (0.56–1.82)	0.984	1.33 (0.78–2.27)	0.296
Always	0.95 (0.56–1.61)	0.836	1.00 (0.63–1.61)	0.993
Blood pressure measurement				
Regular				
Irregular	0.91 (0.55–1.49)	0.724	0.96 (0.59–1.53)	0.859
Knowledge of medication				
low				
high	0.99 (0.68–1.44)	0.974	0.91 (0.63–1.29)	0.581
Intercept	0.248 (0.01–0.2)	<0.001	0.03 (0.01–0.09)	<0.001

**TABLE 4:** The marginal effect of each variable after propensity score weighting.

Variable	*p*	Marginal effect (95%CI)
A usual primary care provider		
Yes		
No	<0.001	0.09 (0.06–0.12)
Gender		
Male		
Female	0.192	0.03 (−0.00 to 0.08)
Age (years)		
18–65		
66–75	0.002	−0.08 (−0.12 to 0.03)
>75	0.921	0.00 (−0.08 to 0.06)
Domicile		
Urban area		
Rural area	0.523	0.02 (−0.04 to 0.08)
Education		
Primary school		
Middle school	0.593	0.01 (−0.03 to 0.06)
High school	0.184	0.04 (−0.02 to 0.10)
University	0.161	0.06 (−0.03 to 0.15)
Job		
Manual worker		
Brain worker	0.048	−0.04 (−0.08 to 0.00)
Residence status		
Live alone		
Not live alone	0.191	0.04 (−0.02 to 0.09)
Annual income		
0–9,999		
10,000–50,000	0.809	0.01 (−0.04 to 0.06)
>50,000	0.345	0.03 (−0.03 to 0.08)
Number of diseases		
1		
2	<0.001	0.09 (0.05–0.12)
≥3	<0.001	0.40 (0.34–0.45)
Disease history/year		
0–10		
11–20	0.005	−0.06 (−0.10 to 0.02)
>20	0.008	0.06 (0.01–0.11)
Adverse disease reaction		
No		
Yes	0.019	−0.05 (−0.09 to 0.01)
Severity of disease		
Mild		
Moderate	0.150	0.03 (−0.01 to 0.08)
Severe	0.049	0.05 (−0.03 to 0.11)
Medical insurance		
Employee health insurance		
Resident health insurance	<0.001	−0.12 (−0.17 to 0.06)
Hospitalization		
No		
Yes	0.260	−0.02 (−0.06 to 0.02)
Communication frequency		
No		
Rare	0.838	0.01 (−0.05 to 0.06)
Occasionally	0.673	0.01 (−0.05 to 0.08)
Often	0.561	0.02 (−0.04 to 0.08)
Always	<0.001	0.14 (0.06–0.22)
Drink		
Never		
Occasionally	0.254	−0.04 (−0.10 to 0.02)
Often	0.828	0.01 (−0.10 to 0.13)
Always	0.027	0.09 (0.00–0.18)
Smoking		
Never		
Have quit smoking	0.245	0.04 (−0.03 to 0.10)
Smoking	0.105	−0.05 (−0.11 to 0.08)
Diet		
Balance		
Mainly meat	0.030	0.08 (0.00–0.16)
Mainly vegetarian	0.049	0.04 (0.00–0.08)
Exercise		
Never		
Occasionally	0.504	0.02 (−0.04 to 0.08)
Often	0.296	0.03 (−0.00 to 0.09)
Always	0.993	0.00 (−0.05 to 0.05)
Regular blood pressure measurement		
Regular		
Irregular	0.859	0.00 (−0.06 to 0.05)
Knowledge of medication		
low		
high	0.581	0.01 (−0.05 to 0.03)
Intercept	<0.001	—

With regard to the covariates, we found that patients who aged between 65 and 75 years (OR = 0.50, 95%CI: 0.33–0.77, *p* = 0.020), used to be brain workers (OR = 0.67, 95%CI: 0.45–0.99, *p* = 0.048), have been sick for 10–20 years (OR = 0.56, 95%CI: 0.37–0.83, *p* = 0.005), have had adverse drug reactions (OR = 0.64, 95%CI: 0.45–0.93, *p* = 0.019), and purchased resident health insurance (OR = 0.35, 95%CI: 0.21–0.58, *p* < 0.001) were less likely to have polypharmacy behaviors. But patients who have two kinds of diseases (OR = 3.05, 95%CI: 1.87–5.10, *p* < 0.001) and above (OR = 21.03, 95%CI: 12.83–35.65, *p* < 0.001), have been sick for 20 years and above (OR = 1.66, 95%CI: 1.14–2.42, *p* = 0.008), with serious diseases (OR = 1.63, 95%CI: 1.00–2.67, *p* = 0.049), communicated with doctor frequently (OR = 3.14, 95%CI: 1.62–6.19, *p* < 0.001), with alcoholism (OR = 2.14, 95%CI: 1.08–4.19, *p* = 0.027), have a meat-based diet (OR = 1.42, 95%CI: 1.00–2.00, *p* = 0.049), and have a vegetarian-based diet (OR = 1.42, 95%CI: 1.00–2.00, *p* = 0.049) were more likely to have polypharmacy behaviors.

## Discussion

### The Effect of Having a Usual Primary Care Provider on Polypharmacy Behaviors

This study provided evidence that patients who did not have a usual primary care provider were more likely to have polypharmacy behaviors than those who had a usual primary care provider (OR = 2.40, 95%CI: 1.74–3.32, *p* < 0.001), with a marginal effect of 0.09 (95%CI: 0.06–0.12), which means that at the average level of other conditions, the probability of polypharmacy behaviors increased by 9.0% when patients who had a usual primary care provider changed to not having a usual primary care provider. In China, medical staff in primary medical institutions have more time to communicate with patients compared to the medical staff in general hospitals. Patients had established a close relationship with primary medical institutions so that they were more willing to seek the guidance of doctors from primary medical institutions in their daily lives (Starfield et al., 2005; Kruk et al., 2010; Feng et al., 2016). In addition, doctors in primary medical institutions are more familiar with patients, which reduces the enthusiasm of patients to seek doctors in advanced medical institutions and reduces the probability of doctors' repeated prescriptions due to untimely sharing of patient's information by patients (Zhang et al., 2013; Duckett et al., 2016).

Although China has established a hierarchical medical system for a long time, many patients go directly to general hospitals instead of primary medical institutions, which may be related to the unbalanced allocation of medical resources (Zhou et al., 2021). At present, there are fewer specialized medical staff in primary medical institutions (Chen et al., 2021), a higher turnover rate of grassroots medical staff (Dale et al., 2015), lower education (Chen et al., 2021), poor medical equipment, and lack of conventional drugs, which makes patients have low trust in primary medical institutions and reluctant to go to primary medical institutions.

With a study showing that patients with primary care providers are likely to reduce patient self-referrals in rural China's rural multi-tiered medical system (Feng et al., 2017) and another study showing that general practitioners (GPs) can effectively reduce patient referral behavior, health maintenance organizations (HMOs) in the United States require patients to consult a primary care physician firstly before considering a referral (Sekhri, 2000; Hoel et al., 2021). Many European countries have GP systems that can effectively guide patients through graded care (Verhaak et al., 2004; Brown et al., 2014).

We need to give play to the role of primary medical institutions. On the one hand, we need to strengthen the construction of primary medical institutions and ensure the complete resources of personnel, equipment, drugs, and other resources in primary medical institutions, so as to restore the trust of patients in primary medical institutions (Liu et al., 1996; Yip and Hsiao, 2014; Shi et al., 2015; Duckett et al., 2016). On the other hand, we need to strengthen the active health management role of primary medical institutions for patients with chronic diseases; establish a community comprehensive medical team including doctors, pharmacists, nurses, and other professionals; and actively manage the diseases of patients in the local area to regain patients' confidence to primary medical institutions (Li et al., 2020; World Bank Group, 2021). In addition, some patients with chronic diseases do not realize the impact of national policies on the improvement of primary medical institutions; we need to strengthen the publicity of the improvement of primary medical institutions to patients, so that patients can understand and trust the medical service capability of new primary medical institutions and to have a usual primary care provider.

### The Effect of Demographic and Clinical conditions on Polypharmacy Behaviors

With regard to the demographic and disease-related factors, patients with more diseases, severe disease, and disease history of more than 20 years are more likely to have polypharmacy behaviors. Patients aged 65–75 years, used to be brain workers, with disease history between 10 and 20 years, had adverse drug reactions, and insured by urban and rural residents' medical insurance were less likely to have polypharmacy behaviors.

The younger patients were associated with polypharmacy behaviors, which is consistent with existing studies (Dwyer et al., 2010; Olsson et al., 2010; Bronskill et al., 2012; Ruths et al., 2013; Nørgaard et al., 2017). The incidence of polypharmacy behaviors decreases with age, which may be related to the increased focus on discontinuing unnecessary medications in older adults with limited life expectancy (Holmes, 2009; Onder et al., 2012).

It is well known that patients with more diseases are more likely to have polypharmacy behaviors (Dwyer et al., 2010), but this study shows that the incidence of multiple drugs' exponential growth as the disease increased should be noted. For patients with chronic diseases, the marginal effect increased by 0.09 from having one disease to having two diseases, indicating a 9.0% increase in the incidence of polypharmacy behaviors. For patients with chronic diseases, the marginal effect increased by 0.40 from having one disease to have three or more diseases, indicating that when other variables were kept at the average level, changes in the number of cases from 1 to 3 or more resulted in a 40.0% increase in the incidence of polypharmacy behaviors.

Patients with a disease history between 11 and 20 years have a lower incidence of polypharmacy behaviors, while patients with a disease history of 21 years and above have a higher incidence of polypharmacy behaviors, which may be related to the health awareness and disease situation of patients with chronic diseases. Patients with a disease history of 11–20 years are aware of the physical damage caused by taking multiple drugs, so they will consciously reduce unnecessary drugs. But patients who had been ill for 21 years or more had to take multiple drugs for their poor health.

Brain workers were less likely to have polypharmacy behaviors, which may be related to the greater job pressure of physical workers (Tan et al., 2020). The incidence of polypharmacy behaviors was lower in patients with adverse drug reactions, which may be because patients with chronic disease are afraid of adverse drug reactions and become more careful in drug use, resulting in a lower incidence of polypharmacy behaviors.

### The Influence of Medical Treatment and Health Behavior on Polypharmacy Behaviors

The incidence of polypharmacy behaviors is higher among patients who participated in urban employee medical insurance. Most of the patients who buy urban employee medical insurance are in economically developed areas; they have access to more sources of drugs, such as pharmacies, hospitals, and primary medical institutions. Therefore, polypharmacy behaviors are prone to occur. In addition, some patients who purchase urban employee medical insurance even have designated cooperative hospitals; it has been shown that patients who frequently visit hospitals have a higher incidence of polypharmacy behaviors than those who tend to visit primary medical institutions.

Patients who had frequent communication with their physicians had a higher incidence of polypharmacy behaviors; doctor–patient shared decision-making has been promoted in recent years, which can effectively improve patient compliance, patient satisfaction, and curative effect and make more beneficial choices for patients (Légaré et al., 2014; van Hoorn et al., 2016; Spatz et al., 2017). Doctor–patient shared decision can promote effective communication between doctors and patients to make medical decisions, rather than increasing the frequency of doctor–patient communication. The frequency of communication between doctors and patients indicates that patients may be treated in multiple departments or places, resulting in repeated prescriptions or prescription cascades that will increase the incidence of polypharmacy behaviors.

The incidence of polypharmacy behaviors is higher in patients with alcoholism. Previous studies have confirmed that moderate alcohol consumption has certain effects on control of chronic diseases (Ford et al., 2012; Loef and Walach, 2012; Zhu et al., 2019; Minzer et al., 2020), but alcoholism was associated with diseases such as cancer, hypertension, and liver disease, so patients who drink frequently were more likely to suffer from more diseases; polypharmacy behaviors were also more likely to happen. It was found that patients who have a meat-based diet or vegetarian-based diet are more prone to polypharmacy behaviors compared to patients who have a balanced diet. Patients with a meat-based diet are more prone to be diagnosed with hypertension, hyperlipidemia, diabetes, and other chronic diseases, so they are more prone to have polypharmacy behaviors happen, and the vegetarian-based patients may have lower body immunity and are prone to disease. In addition, some patients are recommended by doctors to use dietary therapy and other drug substitution therapy to treat chronic diseases, so patients with balanced meat and vegetable may use dietary therapy instead of drug therapy and take fewer drugs (Morgan et al., 2004; Hoel et al., 2021).

According to the findings in this study on the impact of patients' health behaviors and medical treatment behaviors on polypharmacy behaviors, corresponding intervention measures can be taken to prevent polypharmacy behaviors. For the patients who purchase urban employees' medical insurance, we should strengthen the publicity of rational drug use knowledge to avoid polypharmacy behaviors due to their exposure to multiple drug sources. Moreover, we should encourage effective communication between doctors and patients, instead of frequent communication. We should also encourage patients to use alternative therapies such as dietary therapy instead of drug therapy to reduce the possibility of polypharmacy behaviors, while encouraging patients to maintain a reasonable diet and moderate alcohol consumption is also very important to control polypharmacy behaviors.

## Strengths and Limitations

Our study has several limitations. Various definitions of polypharmacy existed in the literature; we only considered the number of drugs used, namely ≥5 drugs as polypharmacy, so it is difficult to make a distinction between necessary prescribing and polypharmacy medication. Secondly, patients were recruited to search for samples, and patients who were unwilling to participate in the study were not investigated, which may lead to certain data bias. Third, since not all patients can remember all the medications, an underestimation of polypharmacy behaviors could not be completely ruled out.

Despite these limitations, this is a rare study in China that explores the impact of having a usual primary care provider on polypharmacy behaviors. Second, we used the propensity score weighting method to adjust for the observed difference in characteristics between those who have a usual primary care provider and those who do not. Third, the data we analyzed come from a large number of patients with hypertension or diabetes equally distributed throughout the 12 regions involved, which makes these evidences characteristic of and comparable to all patients with chronic diseases in Hubei province, China.

## Conclusion

This study provides evidence that patients who had a usual primary care provider had a lower risk of polypharmacy. As we all know, in primary hospitals, the medical staffs have more time to communicate with patients and have a closer relationship with the patients; therefore, patient could have access convenient and economical services and obtain more guidance about drug use. Moreover, with the implementation of the hierarchical diagnosis and treatment and family physician system, the role and function of the primary care hospital in the system are providing integrated service for local residents. In consequence, the healthcare government should make efforts to construct community level medical institutions and improve the quality of health service to attract more patients, especially those with hypertension and diabetes.

## Data Availability

The data analyzed in this study are subject to the following licenses/restrictions: Because the data involve the patient's personal information, it cannot be provided. Requests to access these datasets should be directed to fengda@hust.edu.cn.
